# Selecting, Scaling, and Measuring the Value of Ambient AI in a Nonacademic Health System: Multiphase Pilot Study

**DOI:** 10.2196/87450

**Published:** 2026-06-26

**Authors:** Bryon Kenneth Frost, Victor Eugene Collier, Franklin Sturgill, Jessie Polson, Jennifer Jones

**Affiliations:** 1 Department of Information Technology, McLeod Health, 555 E. Cheves Street, Florence, SC, 29501, United States, 1 843 777 5464; 2 Department of Internal Medicine, McLeod Health, Florence, SC, United States

**Keywords:** artificial intelligence, AI, clinical documentation, health system implementation, workflow integration, physician burnout

## Abstract

**Background:**

Most US health systems operate on a local or regional scale and face substantial financial and staffing pressures, which are intensified by challenges related to physician satisfaction and difficulties in recruitment and retention. Ambient artificial intelligence (AI) documentation solutions have the potential to reduce burdens and improve satisfaction, but vendor selection is often undermined by cognitive biases, unvalidated marketing claims, and limited real-world testing.

**Objective:**

To address this, McLeod Health developed and implemented an objective, multiphase approach to evaluate and adopt an ambient AI solution across its multihospital system.

**Methods:**

Our evaluation process began in spring 2024 with 4 leading vendors tested through live clinical simulations using 15 complex outpatient scripts, with organizational leaders serving as standardized patients. Ambient patient encounters were captured in real time, and AI-generated notes were scored by physicians, revenue cycle experts, and nonclinical reviewers for accuracy, billing quality, and readability. The top 2 vendors advanced to demonstrations of Epic workflow integration, with physician usability feedback guiding the final selection. In the third phase, the chosen vendor underwent a 90-day pilot across 5 ambulatory specialties beginning in October 2024, followed by system-wide implementation in January 2025. Key performance indicators included documentation time, coding, and financial trends, as well as patient and provider satisfaction. All statistical comparisons were 2-sided using a 95% CI.

**Results:**

The 3-phase evaluation process resulted in careful vendor selection. The pilot showed a 35.4% decrease in pajama time (*P*=.054, trend toward significance) and a 28.3% decrease in time in notes (n=23; *P*<.001). Coding patterns shifted toward higher-complexity visits, with a 3.8% increase in level 4 established patient visits (*P*=.05), and established patient volumes increased by 8.5%, which was associated with a projected revenue gain of US $2629 per provider per month. Patient satisfaction improved significantly across multiple domains, with large gains in listening, trust, communication, and treatment information (all *P*<.001). These gains exceeded those of prior system-wide patient satisfaction initiatives. System-wide rollout has achieved 81% adoption, with more than 150,000 notes generated.

**Conclusions:**

Our structured, multiphase evaluation process minimized vendor influence and cognitive bias during selection, validated results through real-world clinical testing, and enabled a system-wide rollout. This approach offers a practical framework for nonacademic health systems to objectively assess, implement, and scale ambient AI solutions while preserving fairness, transparency, and measurable value.

## Introduction

### Background

Most health systems in the United States are nonacademic, operate on a local or regional scale, and face far greater pressures to deliver care than well-resourced academic medical centers (AMCs) or private multistate conglomerates. These pressures are especially acute in rural and critical access hospitals, where thin margins and physician burnout make recruitment and retention difficult. Of the 639 health systems across the country, 84% (n=537) operate in a single state with 399 beds, 2 hospitals, and 339 physicians; most are nonteaching hospitals and have a median net hospital revenue of US $2 billion [1].

Ambient artificial intelligence (AI) solutions have the potential to reduce documentation burden, improve physician and patient satisfaction, and increase revenue capture—all of which are critical to the sustainability of health systems delivering most US health care [2-5]. However, nonacademic health systems typically lack the resources of AMCs or large private systems to evaluate how these technologies will perform in their environments [6,7]. The literature calling for more research, especially on the economic and system-level burden of this technology, has gained momentum [8]. Selecting and integrating ambient AI solutions into daily clinical workflows is complex and highly vulnerable to cognitive bias during the selection process [9,10]. Moreover, despite frameworks for assessing ambient AI tool quality and examples of system-wide adoption by large conglomerates, there is no standardized process for the majority of health systems to systematically evaluate, pilot, and scale these solutions [7,11,12].

McLeod Health is a nonprofit, nonacademic health system comprising 7 hospitals and more than 1200 providers serving 18 counties in North and South Carolina. While larger than the median US health system, McLeod operates without the dedicated research infrastructure, centralized innovation programs, or academic funding streams typical of major AMCs. Its scale and decentralized clinical operations place it within a broad group of mid-sized, community-based systems that must balance technology adoption with financial stewardship, clinician workload, and operational constraints.

This paper describes the objective, multiphase approach we used at McLeod Health to select and implement an ambient AI solution. McLeod Health reflects the structure and constraints of nonacademic regional health systems across the country more so than AMCs, which have reported much of the current ambient implementation experience, making our experience broadly relevant. By detailing our evaluation process and outcomes, we offer practical lessons that can guide other nonacademic health systems in adopting and scaling ambient AI technologies.

### The Main Objective

The purpose of this project was to create and implement a structured, multiphase process for selecting an ambient AI solution that minimized vendor influence and cognitive bias in vendor selection, relied on real-world clinical testing, and measured outcomes across key domains: documentation efficiency, physician satisfaction, patient satisfaction, and financial return on investment.

## Methods

### The Team

All phases of this project were conducted across the McLeod system. The initiative was led by the chief medical informatics officer and supported by a broad leadership team that included system and regional chief medical officers, ambulatory practice leadership (senior vice president, chief medical officer, chief operating officer, and advanced practice providers), the chief information officer, and IT leadership, the directors of revenue integrity, patient satisfaction, marketing, clinical informatics, the hospital chief of staff, multiple physician champions, and nursing leadership. This multidisciplinary and interprofessional structure ensured that evaluation, selection, and implementation decisions reflected the perspectives of clinical, operational, financial, and informatics stakeholders across the health system.

### The Evaluation

**Figure 1.**
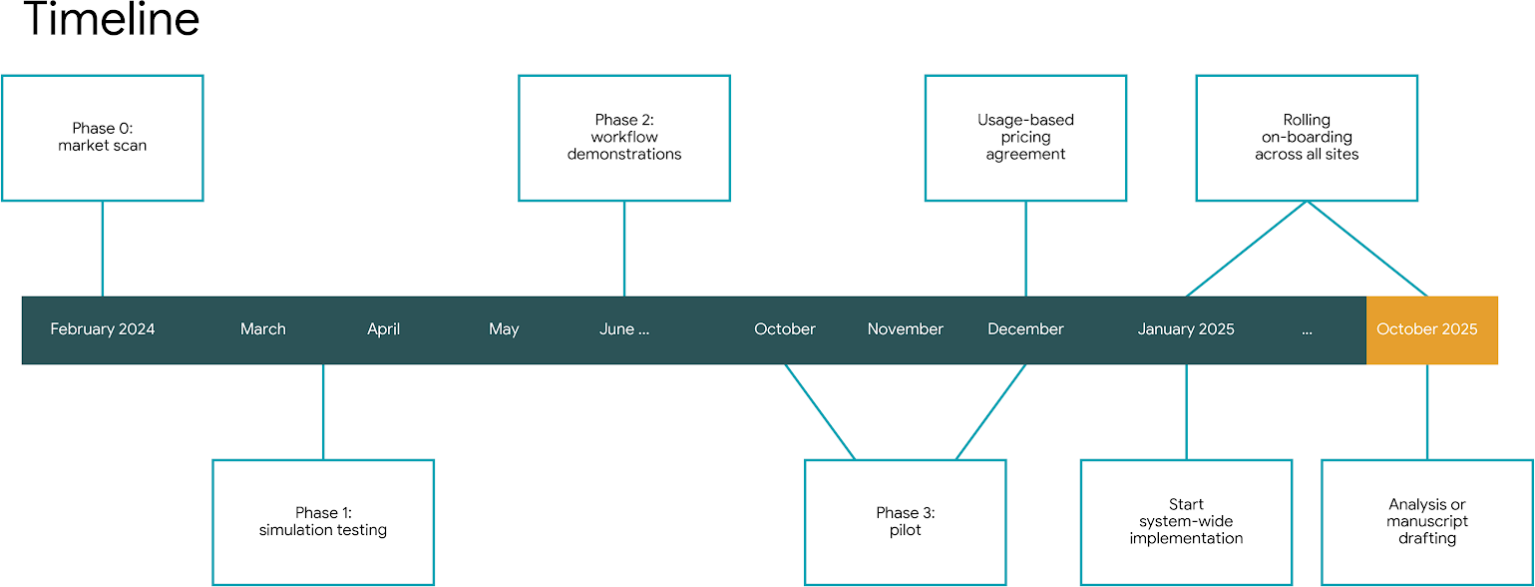
Timeline of pilot and implementation.

We developed a systematic, multiphase approach to objectively evaluate vendor performance, usability, and integration in a large nonacademic health system. The design began in February 2024 (phase 0), with phases 1 and 2 conducted from March through June 2024. A 90-day pilot (phase 3) began in October 2024 (Figure 1).

#### Phase 0: Market Scan

We identified potential vendors by reviewing “Ambient Speech” listings on KLAS and “Digitally Assisted Provider Documentation” vendors on the AVIA marketplace, both of which are trusted by the health care executive community as unbiased sources of data on the industry [13,14]. Vendors were excluded if they raised concerns regarding data security, financial stability, or scalability. Preference was given to those with product roadmaps extending beyond documentation to broader workflow enhancements. Four vendors met these criteria and advanced to phase 1.

#### Phase 1: Simulation Testing

Four vendors participated in a live patient simulation at McLeod Health. We anticipated that 3 main groups of clinicians would most likely benefit from ambient AI in practice: generalists, medical specialists, and surgical specialists. We selected these 3 specialties because they represent the breadth of medical practice and carry a high documentation burden with cognitively complex visit types, making them the most likely to realize meaningful benefits from ambient documentation. To represent these categories, we selected family medicine, cardiology, and general surgery. In collaboration with a revenue cycle expert, we developed the 5 most common patient encounters for each specialty, creating 15 standardized scripts. The cases incorporated real-world challenges such as difficult accents, contradictory family input, and interruptions to mimic the complexity of clinical practice.

Clinical staff and hospital leadership served as actors portraying patients. They were trained using scripts and could modify their cases to add variability and realism. Three McLeod physicians, representing family medicine, cardiology, and general surgery, conducted the simulated encounters. Each physician reviewed the cases in advance and carried out 5 simulated patient visits.

Simulations took place over several hours on 2 days in a large conference room to ensure transparency. Vendors sat in the front row and were required to record the encounters in real time using their solutions. They submitted the resulting notes immediately to the chief medical informatics officer without any opportunity for edits. Three vendors participated in person, while the fourth was evaluated separately using recordings of the cases. Notes were formatted as plain text and labeled only with the scenario patient name and number. Vendor identity was removed before distribution to the evaluators. Evaluators scored notes independently and remained unaware of which vendor generated each set throughout the review process.

A panel of 25 evaluators attended the sessions. The group included representatives from operations, clinical practice, revenue cycle, and patient satisfaction. Evaluators received the notes at the end of the week and were blinded to the vendors. They were instructed to review the notes, rank vendors based on quality, and disregard any marketing information they had received. Rankings were submitted individually, and evaluators did not have access to each other’s scores. Points were assigned as follows: 3 points for the first choice, 2 points for the second choice, and 1 point for the third choice. Results were tabulated and presented to health system leadership for review.

#### Phase 2: Workflow Demonstrations

The 2 top-ranked vendors from Phase 1 advanced to the second phase. Each vendor was required to demonstrate how its solution would integrate with Epic in a simulated clinical encounter. The demonstration included coding support and real-time note generation for a predetermined complex outpatient case. The chief medical informatics officer acted as the simulated patient to ensure a standardized clinical encounter across both vendor demonstration days. Vendors functioned as the clinician in real time, conducting the interview and demonstrating how documentation would flow into Epic.

Approximately 30 physicians from across the health system participated as observers. They represented nearly all major specialties, except for psychiatry and pediatrics, as well as some smaller subspecialties. After the demonstrations, the physicians were asked to vote for 1 vendor, and to ensure fairness, only physicians who attended both demonstrations were permitted to vote. Their assessments were based on acceptability, workflow fit, and the potential impact on daily practice. At the same time, clinical informatics and IT security teams independently assessed each vendor. They examined the technology stack, implementation roadmap, and data safety, along with each vendor’s capabilities beyond documentation.

When the results were collated, both physicians and technical reviewers reached the same conclusion. One vendor’s product clearly outperformed the other, receiving overwhelming support from the physician observers and positive assessments from the informatics and information technology teams. The health system leadership had initially planned to conduct a side-by-side trial in phase 3. However, given the consistent findings, the leadership team canceled this planned trial and advanced the preferred vendor to the pilot phase.

#### Phase 3: Pilot Testing

The third phase was a 90-day pilot across multiple ambulatory specialties, including obstetrics and gynecology, orthopedic surgery, oncology, primary care, and emergency medicine. The health system initially purchased 500 licenses. To manage costs and ensure consistent use, participation was limited to physicians within the top 30% for documentation efficiency who were not already using human scribes. This selection intentionally favored experienced users to establish a reliable baseline and maximize the likelihood of measurable outcomes. Health system leadership emphasized that any efficiency gains should be applied to reducing after-hours documentation rather than increasing patient volume, to avoid exacerbating physician burnout.

Our primary objective in phase 3 was to assess the clinical effectiveness of the tool rather than its adoption. We assumed that if the solution delivered meaningful value, adoption would follow. In addition to pilot physicians, licenses were made available to selected administrative and inpatient clinicians for organizational visibility; however, these individuals were not intended to be part of the pilot cohort and were not included in outcome analyses. Our evaluation focused on ambulatory clinicians who incorporated the tool into routine outpatient practice. To avoid dilution effects, we required more than 20% utilization across encounters, 3 months of baseline data, and at least 1 month of active use. This approach allowed us to evaluate performance among clinicians who meaningfully integrated the technology into their workflow.

Key performance indicators included documentation efficiency, financial impact, and both provider and patient satisfaction. Adoption was defined as completing onboarding and actively using the solution to generate notes at 30 days and again at 5 to 8 weeks after onboarding. Measured documentation metrics included time spent documenting after work hours (“pajama time”), average time in notes per encounter, and the same-day note closure rate using Epic Signal data and established definitions.

Financial impact was modeled by evaluating coding distributions for established patients before and after implementation. Coding charges were based on the evaluation and management codes documented by each provider before and after the start of the pilot. Encounters were grouped by evaluation and management level, and published Centers for Medicare and Medicaid Services reimbursement values were applied to estimate potential changes in revenue [15]. This analysis was limited to established patients, where documentation-related shifts in coding were expected to be most evident. This analysis was also part of the standard evaluation process between McLeod and the vendor and was reviewed collaboratively on a regular basis. There were no usage fees during the pilot. A co-author (DS) independently verified and corroborated all vendor-supplied data through direct extraction of Epic Signal documentation metrics, along with charges, revenue value units, and scheduling data from the electronic health record (EHR). No discrepancies were identified.

Patient satisfaction was assessed using National Research Corporation Health survey questions for each provider over 8 months before and after the pilot (Table 1). The baseline period was February 2024 through September 2024; the pilot period was October 2024 through December 2024; the postpilot period was January 2025 through August 2025. This is a standardized process for the institution, consisting of 3 attempts to contact patients, with 24 hours between attempts: first by SMS text messaging, second by email, and third by interactive voice response phone call. Scores for the Patient Experience survey are calculated as “Percent Positive.” This is determined by dividing the number of patient responses in the “Top Box” by the total number of surveys administered per time period. Responses in the Top Box were defined as “Yes, definitely” (from the options “No,” “Yes, somewhat,” “Yes, mostly,” and “Yes, definitely”) and ratings of 9 or 10 on a scale from 0 (“lowest”) to 10 (“highest”). This methodology represents a standard approach for assessing patient experience scores over time. Scores were compared with the same provider’s results from the prior year in a matched-pair analysis. In addition, providers completed a 7-question survey 1 month after the pilot to assess their ability to schedule more patients, their average time spent writing notes, and their overall satisfaction with the solution. For all comparative analyses, statistical tests were 2-sided.

To determine success in phase 3, we used a pragmatic risk and benefit assessment rather than fixed quantitative benchmarks. We weighed direct vendor costs and internal support requirements against provider-reported usability and perceived reduction in documentation burden. The pilot was considered successful when clinical value and provider acceptance justified the financial and operational investment.

**Table 1.** National Research Corporation (NRC) survey questions to assess patient satisfaction.

Abbreviation	Full survey question	Answer options
Provider listened	Did this provider listen carefully to you?	“No” “Yes, somewhat” “Yes, mostly” “Yes, definitely”
Rating of provider	Using any number from 0 to 10, where 0 is the worst provider possible and 10 is the best provider possible, what number would you use to rate this provider?	0 (“Worst provider”) to 10 (“Best provider”)
Trust provider w/ care	Did you trust this provider with your care?	“No” “Yes, somewhat” “Yes, mostly” “Yes, definitely”
NPS^a^: provider would recommend	How likely would you be to recommend this provider to your family and friends?	0 (“Not at all likely”) to 10 (“Extremely likely”)
Got enough information re: treatment	Did this provider give you enough information about your health and treatment?	“No” “Yes, somewhat” “Yes, mostly” “Yes, definitely”
Knew what to do if questions	Did you know what to do if you had more questions after your visit?	“No” “Yes, somewhat” “Yes, mostly” “Yes, definitely”

a NPS: net promoter score.

### Ethical Considerations

This project was reviewed by the McLeod Health Institutional Review Board and formally determined not to constitute human subjects research per 45 CFR 46.102 [16]. It was characterized as a quality assessment and quality improvement initiative. A formal institutional review board review was not required.

## Results

### Phase 1

All 25 evaluators submitted note quality rankings within 2 weeks, producing a 100 percent response rate. Of these, 17 submitted complete 3-choice rankings, while 8 submitted a first-place preference only. Based on 45 notes, the overall scores were: vendor 1 with 57 points (45.2% of the vote), vendor 2 with 44 points (34.9%), and vendor 3 with 25 points (19.8%). When the physician subgroup rankings were reviewed, they mirrored the overall results, with vendor 1 receiving 45.8%, vendor 2 receiving 37.5%, and vendor 3 receiving 16.7%. The fourth vendor’s notes were reviewed but excluded from further analysis due to noncompetitive pricing and contractual infeasibility.

### Phase 2

Physician evaluations and independent technical reviews by the informatics and IT security teams reached the same conclusion. Nearly 20 physicians who observed both demonstrations overwhelmingly preferred vendor 2, with a 90 percent favorability rating compared to vendor 1. Technical reviewers also favored vendor 2, citing a more advanced technology stack and broader functionality beyond documentation, including conversational command capabilities within the EHR, specialty-specific chart summarization, and voice-guided billing and coding assistance. Although vendor 1 received higher note quality scores in phase 1, vendor 2 was rated higher in phase 2 because these additional capabilities were viewed as strategically important for workflow integration and long-term scalability. Based on the alignment between physician and technical reviewers, health system leadership determined that a side-by-side trial was unnecessary and advanced vendor 2 to the pilot phase.

### Phase 3

During the 3-month pilot, 49 clinicians were onboarded in 2 cohorts. The adoption rate during the pilot was 79.6% (n=39). Based on the previously described analysis criteria, data from 23 providers were analyzed. The specialty breakdown was as follows: family medicine (n=12, 52.2%), orthopedics (n=5, 21.7%), oncology (n=4, 17.4%), and emergency medicine or urgent care (n=2, 8.7%). Of the analyzed providers, 70% were physicians (DO or MD), and 30% were advanced practice providers (physician assistant, nurse practitioner, and advanced practice registered nurse). Participants from obstetrics and gynecology did not meet the inclusion criteria for analysis.

Documentation efficiency improved substantially. Pajama time decreased by 35.4% per provider per 4-week period, with a trend toward significance (*P*=.054). Time spent on notes decreased by 28.3% (*P*<.001), saving 3.6 hours per provider per month, or 83 hours across the group. Same-day note closure did not change significantly.

**Figure 2.**
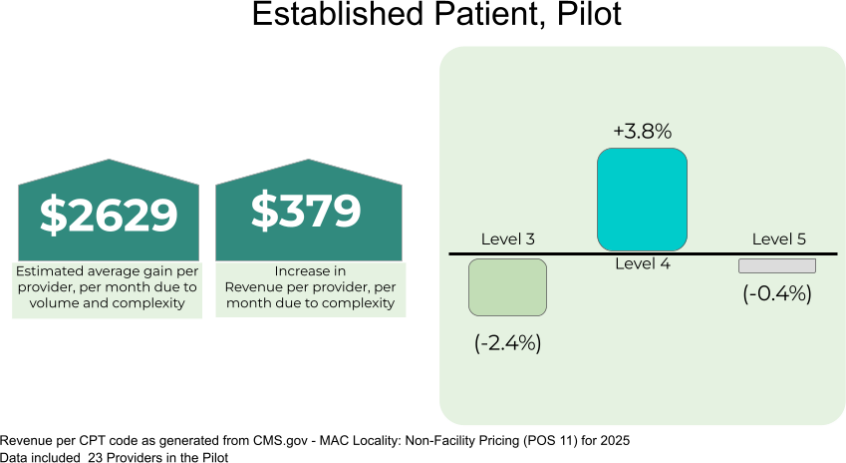
Estimated changes in coding and revenue after the 90-day pilot of an ambient artificial intelligence solution, using the Center for Medicare and Medicaid Services nonfacility Place of Service 11 pricing. POS: Place of Service.

Coding patterns shifted toward higher complexity for established patients. Level 3 visits decreased by 2.4% (*P*=.17), level 4 visits increased by 3.8% (*P*=.05), and level 5 visits decreased slightly by 0.4% (*P*=.66). These coding shifts alone produced an estimated average gain of US $379 per provider per month (Table 2). When combined with the 8.5% increase in established patient volume, the overall average projected revenue gain was US $2629 per provider per month, representing the total estimated impact of both coding complexity shifts and volume increases (Figure 2). All percentage change results are based on unrounded raw medians. We cannot exclude seasonal variation as a contributing factor, as a concurrent control group was not available. The pilot results were presented to senior leadership and contributed to the decision to scale the solution system-wide.

**Table 2.** Key efficiency and revenue metrics at McLeod Health before and after the use of an ambient artificial intelligence solution.

Variable	Pilot (n=23)			
	Pre	Post	Change (%)	*P* value
Efficiency metrics, median (IQR)				
Median pajama time per 4-week period (h)	5.6 (2.2-9.4)	3.6 (1.0-8.6)	−35.43^a^	.054
Median time in note per appointment (min)	2.8 (1.9-4.4)	2.0 (1.1-3.4)	−28.34^a^	<.001
Median same-day note closure rate (%)	68.0 (35.6-87.5)	69.8 (39.8-94.2)	1.15^a^	.43
Median time on unscheduled days (h)	5.4 (1.2-7.7)	2.2 (0.6-7.8)	−59.78^a^	.09
Evaluation and management codes (%), mean (SD)				
Average level 3 established encounters (99213)	28.5 (19.2)	26.1 (21.3)	−2.40^b^	.17
Average level 4 established encounters (99214)	60.9 (18.6)	64.7 (20.5)	3.77^b^	.05
Average level 5 established encounters (99215)	7.1 (14.8)	6.7 (14.2)	−0.39^b^	.66
Estimated revenue for established patient care, mean (SD)				
Average increase in established patient volume	223.3 (96.0)	242.3 (110.6)	8.50^b^	.002
Due to increased complexity (US $)	—^c^	—	379 (48)^b^	—
Due to increased volume and complexity (US $)	—	—	2629 (1892)^b^	—

a % change.

b % difference.

c Not applicable.

**Figure 3.**
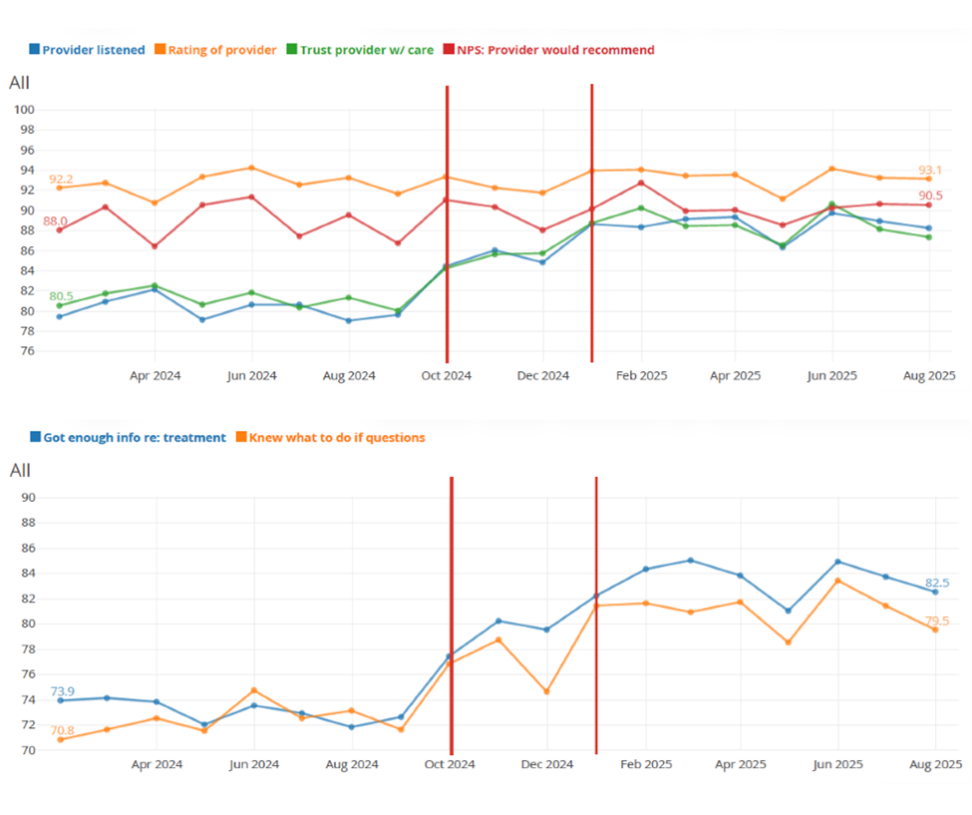
Patient satisfaction survey results for the pilot at McLeod Health (n=21, median response rate of 32.7% or 1377 responses per month, courtesy of ©NRC Health. (2026). *Experience improvement database- medical practice*. February 2024- August 2025. [Dataset] NRC Health [17]; NPS: net promoter score.

Median patient satisfaction scores were compared between the 8-month baseline period (February 2024 to September 2024) and the 8-month postpilot period (January 2025 to August 2025) for 21 of 23 providers based on data availability. Scores increased significantly, reflecting sustained improvement that extended well beyond the initial 90-day pilot window. Median scores increased from baseline for: “Provider listened” (8.4 points; *P*<.001), “Trust in provider” (7.5 points; *P*<.001), “Knew what to do if they had questions” (8.8 points; *P*<.001), and “Received enough information regarding treatment” (10.4 points; *P*<.001; Figure 3). Ratings for “Provider would recommend,” and overall provider rating remained high without significant change (1.6 points; *P*=.09 and 0.7 points; *P*=.28, respectively). The median response rate was 32.7% across the entire period (n=1377 responses per month, or n=66 per provider). The median response rate was not statistically different before and during the pilot (34.1% vs 33.8%; *P*=.23), and during and after the pilot (33.8% vs 32.9%; *P*=.81) for the 21 providers.

Provider experience was assessed with a survey administered 1 month after the pilot. Eighteen physicians responded (36.7% response rate, n=49). Half reported being able to schedule or see more patients per shift, and 89% reported spending 5 minutes or less per note. The median satisfaction score was 8 out of 10, accuracy was 7.5, and all respondents rated their likelihood of recommending the solution to a colleague as 10 out of 10.

The pilot met our criteria for success, and the hospital leadership decided to proceed with a larger-scale rollout.

### System-Wide Implementation Progress

Interest in the ambient AI solution grew quickly during the pilot as physicians and staff observed improvements in efficiency, provider well-being, patient satisfaction, and financial performance. This demand created pressure to expand access beyond the pilot group. To address cost concerns and support broader use, health system leadership negotiated a new financial model with the vendor that shifted to usage-based pricing with a capped monthly rate. This structure reduced the risk of unused licenses and aligned incentives between the health system and the vendor.

Once the new model was in place, preliminary results were shared in a system-wide letter with approximately 675 ambulatory clinicians. Access was then expanded to all interested physicians and advanced practice providers who were not using human scribes, regardless of their efficiency. To meet anticipated demand, the health system purchased additional licenses to further expand access to inpatient teams.

Onboarding is occurring in phases to support workflow adaptation. The decentralized nature of McLeod’s clinics and facilities has made communication challenging, so leadership launched an educational campaign that included physician testimonial videos highlighting early successes. In March 2025, self-onboarding was introduced to simplify adoption further.

**Figure 4.**
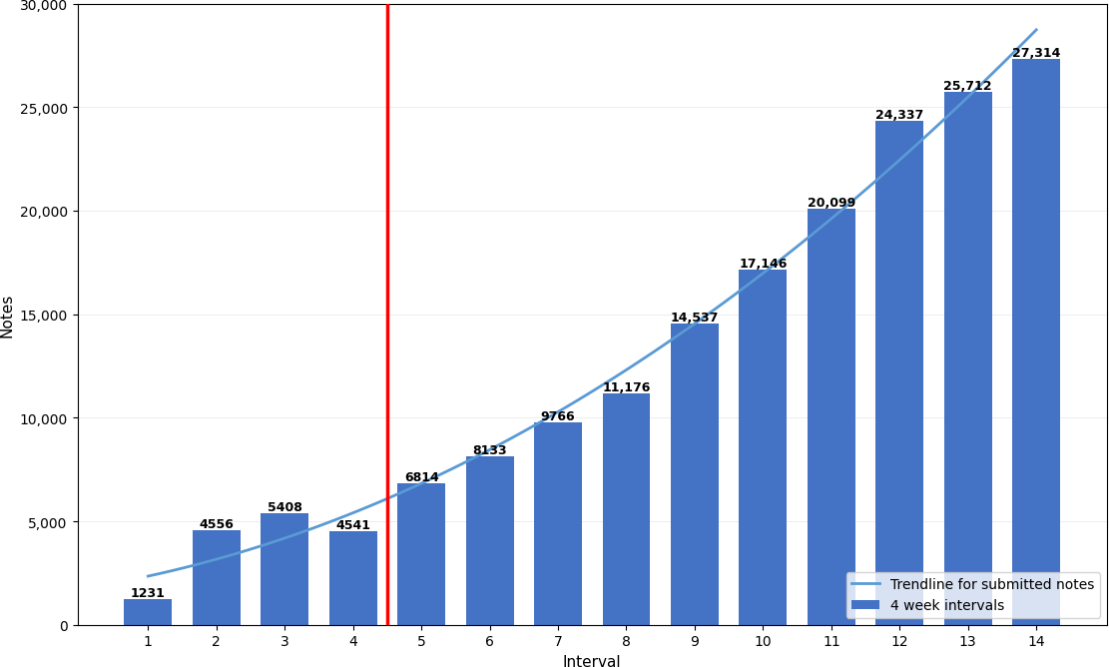
Note volumes in 4-week intervals since the start of the pilot at McLeod Health (red line indicates the transition from pilot to system-wide rollout with usage-based pricing).

Since the system-wide implementation began in January 2025, 250 providers have been onboarded in phases, achieving an adoption rate of 81%. Collectively, these providers have generated nearly 150,000 notes using the ambient AI solution (Figure 4). Analysis is in progress, but early indications are consistent with the results of the pilot, demonstrating improved documentation time and coding shifts.

## Discussion

### Principal Findings

Our multiphase evaluation and implementation yielded substantial gains in documentation efficiency, clinician and patient satisfaction, and financial performance, while also generating practical lessons for nonacademic health systems.

While frameworks that assess the quality of various ambient AI tools have been published, we have not found any systematic frameworks designed to guide nonacademic health systems in selecting ambient AI vendors for implementation. We initially assumed that a well-established vendor would best meet our needs. However, our objective evaluation revealed that a lesser-known vendor outperformed a prominent competitor in real-world clinical testing. This demonstrated the importance of a transparent selection process that minimizes bias, secures clinician buy-in, and focuses on actual performance under realistic conditions. In our experience, high-stakes technology decisions in health care are inherently vulnerable to conformity bias, particularly when vendors benefit from strong market visibility or widespread peer adoption. Although we did not directly measure cognitive bias, the evaluation framework was intentionally designed to reduce susceptibility to reputation or consensus-driven selection. Predefined criteria, parallel vendor testing, independent technical and clinical assessments, and reliance on objective workflow metrics were used to anchor decision-making in observed performance rather than sales presentations or peer adoption patterns. This data-driven selection approach may help other health systems, particularly those with limited financial margins, reduce costly misalignment between vendor claims and operational realities.

The benefits of ambient AI extend beyond documentation efficiency alone [4,18-20]. We identified several additional benefits that have not been well-quantified in the medical literature on ambient AI. In our setting, time in notes decreased significantly, established patient volume increased, and coding shifts toward higher complexity were associated with an estimated $2629 per provider per month. Additionally, while this data was part of the standard evaluation process between McLeod and the vendor, to ensure the accuracy of these results, we performed an independent review of the raw data from the electronic record and charges filed during this period and found that the trends remained consistent with these results. After the system-wide rollout, we observed continued and persistent improvements in these operational and financial metrics for high users. For small and mid-sized systems with narrow operating margins, these findings, if replicated in broader provider populations, strengthen the case for investment in ambient AI. Patient satisfaction also improved across multiple metrics in ways that far exceeded prior system-wide initiatives. Though we recognize that correlation does not prove causation and that other secular trends we did not assess could have influenced our findings, the results align with published literature demonstrating improvements in patient satisfaction after the implementation of ambient AI technology [21]. Plausible mechanisms for improvement include focused attention with less keyboard time and a lower perception of distractibility. These gains have also been sustained for months after the pilot, providing longer-term data that remains scarce in the literature.

Our implementation strategy shaped outcomes in unexpected ways. During the pilot, we targeted the top 30 % of providers by efficiency to maximize early adoption and financial return. This conservative approach was driven by concerns over license costs under a subscription model. While the strategy reduced financial risk, it likely underestimated the potential benefits for providers with heavier documentation burdens. However, it remains unclear whether baseline documentation efficiency meaningfully influences adoption patterns or sustained usage, and this question warrants further investigation [22]. We were surprised to see the high levels of adoption in the pilot, but this rate has stayed consistent over the year since system-wide implementation, suggesting that our initial rate was not an overestimate. Negotiating a usage-based pricing model has allowed us to expand access more broadly, aligning vendor and system interests. There is no published evidence identifying which clinicians or specialties benefit most from ambient AI, a gap that should guide future research and implementation strategies.

Scaling across a decentralized health system posed challenges. With numerous clinics and specialty practices spread over a large geographic area, the dissemination of information and consistent adoption strategies were difficult. This experience highlights a structural challenge faced by many nonacademic systems [7]. The primary barrier was not the technology but resistance to changing established documentation habits. Similar to prior transitions from typed documentation to speech recognition, skepticism persisted until clinicians experienced meaningful efficiency gains firsthand. Peer influence proved more effective than formal training. Physicians who demonstrated measurable reductions in documentation time and after-hours work gradually became informal champions, helping normalize adoption across clinics. To support this, we developed an onboarding website with workflow resources and created 12 testimonial videos highlighting early physician experiences. These efforts helped sustain engagement across the organization. This experience may be informative for other health systems implementing ambient technology.

### Comparisons With Prior Work

Our results are consistent with several recent publications and particularly add to the literature for nonacademic health systems. An analysis of five ACMs found that the adoption of ambient AI tools was associated with decreases in total time spent in the EHR and time spent documenting (13.4 and 16.0 min, respectively) [23]. While these are not the same measures that we used, they represent the same trend across documentation efficiency measures. Their effects varied by user type with primary care specialties, users with adoption rates of more than 50%, as well as advanced practice clinicians and female clinicians, showing more gains than others. This corroborates our experience that adoption and value vary across a number of factors that have not yet been studied. Two separate analyses at major ACMs found that ambient AI adoption was associated with a modest increase in relative value units [24,25]. This again reflects the trend seen in our data. A randomized clinical trial comparing different ambient AI tools at an ACM found that clinicians had meaningfully different experiences across tools. This suggests that tool selection should be evaluated at the health system level, as we demonstrate in this paper [26].

### Limitations

First, the limitations of our project include its single-system design, small pilot, lack of control for seasonality or case mix, and absence of a validated burnout assessment. Second, in phase 2, the chief medical information officer served as the simulated patient to ensure a standardized clinical encounter across both vendor demonstrations. Vendor selection was ultimately determined by independent physician voting and technical team assessments conducted without the chief medical information officer’s influence; however, this could have led to some unmeasured bias. Third, in phase 3, we prioritized analysis of providers most likely to demonstrate measurable benefit, and therefore, 16 of 39 active participants were subsequently excluded for not meeting minimum usage thresholds. The final cohort of 23 providers in the pilot represents high-adopting clinicians, and the observed efficiency and financial gains may not generalize to providers with heavier documentation burdens, lower baseline efficiency, or more modest adoption patterns. Fourth, we also did not conduct any internal audits or compliance reviews beyond standard processes during the pilot window. Fifth, for the financial analysis, we were conservative in assessing gains due to increases in volume and complexity of established patients and did not account for gains from new patient volume or complexity due to concern for potential confounders. Additionally, we did not conduct a total cost of ownership analysis, and while there were no usage costs during the pilot, we also did not include cost savings on unused subscriptions from using a usage-based pricing model that was implemented during the system-wide rollout.

### Recommendations

For the next steps, broader studies across multiple sites and specialties are needed to evaluate generalizability and to capture additional benefits, such as improved *International Classification of Diseases, Tenth Revision* (*ICD-10*) coding, hierarchical condition category capture, or closure of care gaps. Clinician satisfaction, coding changes, and burnout should also be tracked over time, as sustained access to ambient AI may reduce attrition and recruitment costs. Patient satisfaction gains, particularly improvements in trust and communication, may also yield indirect financial benefits through value-based care contracts and organizational reputation. Finally, while overall satisfaction was high and reflected in the strong net promoter scores and positive qualitative feedback, clinicians identified areas for improvement in the ambient AI solution, such as enhanced specialty-specific customization, stronger inpatient functionality, and improved adaptability to individual documentation styles. Addressing these needs will be essential for long-term engagement. Despite these challenges, our findings suggest that ambient AI can meaningfully improve efficiency, satisfaction, and financial outcomes in nonacademic health systems.

### Conclusions

In summary, this structured, multiphase framework minimized vendor influence and cognitive bias in the selection process, validated performance through objective clinical testing in a pilot, and guided a successful system-wide rollout at McLeod Health. Our experience demonstrates that nonacademic health systems can select and adopt ambient AI successfully using a transparent, evidence-based process that does not require major academic resources. When effectively integrated, ambient AI can reduce documentation burden, improve satisfaction for both providers and patients, and deliver measurable financial returns, which may be meaningful for other nonacademic health systems across the country.

## Abbreviations

AI: artificial intelligence
AMC: academic medical center
EHR: electronic health record
*ICD-10*: *International Classification of Diseases, Tenth Revision*
